# Clindamycin suppresses virulence expression in inducible clindamycin-resistant *Staphylococcus aureus* strains

**DOI:** 10.1186/s12941-018-0291-8

**Published:** 2018-10-20

**Authors:** Elisabeth Hodille, Cédric Badiou, Caroline Bouveyron, Michèle Bes, Anne Tristan, François Vandenesch, Gérard Lina, Oana Dumitrescu

**Affiliations:** 1 Department of Bacteriology, Hospices Civils de Lyon, Hôpital de la Croix-Rousse, Centre de Biologie Nord, Lyon, France; 2 National Reference Center for Staphylococci, Hospices Civils de Lyon, Hôpital de la Croix-Rousse, Centre de Biologie Nord, Lyon, France; 3 Centre International de Recherche en Infectiologie (CIRI), INSERM U1111, CNRS UMR5308, ENS Lyon, Université Lyon 1, Lyon, France

**Keywords:** *Staphylococcus aureus*, Panton–Valentine leucocidin, Toxic-shock-staphylococcal toxin, Alpha-haemolysin, Inducible clindamycin resistance, Anti-toxin effect

## Abstract

Clindamycin is a protein synthesis inhibitory agent that has the ability to suppress the expression of virulence factors in *Staphylococcus aureus*. Recent guidelines recommend the use of clindamycin for the treatment of toxin-mediated infections. Clindamycin modulates virulence expression at sub-inhibitory concentrations (sub-MICs) in clindamycin-susceptible *S. aureus* strains but previous report shown that this effect was supressed for constitutive clindamycin resistant strains. However, no data are currently available on the impact of clindamycin at sub-MICs on the virulence of inducible clindamycin-resistant *S. aureus* strains. Here, we show that sub-MICs of clindamycin decrease Panton–Valentine leucocidin, toxic-shock-staphylococcal toxin (TSST-1) and alpha-haemolysin (Hla) expression in six inducible clindamycin-resistant isolates cultivated in vitro in CCY medium. These results suggest that the clindamycin anti-toxin effect is retained for inducible clindamycin-resistant *S. aureus* isolates; therefore, its usage *s*hould be considered within the treatment regimen of toxin related infections for inducible clindamycin-resistant *S. aureus*.

## Introduction

Clindamycin is a protein synthesis inhibitory agent that has the ability to suppress the expression of virulence factors in *Staphylococcus aureus* at sub-inhibitory concentrations (sub-MICs). Indeed, several studies have reported the ability of clindamycin at sub-MICs to decrease the production of Panton-Valentine leucocidin (PVL), toxic-shock-staphylococcal toxin (TSST-1) or alpha-haemolysin (Hla) [1–6]. Therefore, recent guidelines recommend the use of clindamycin for the treatment of toxin-mediated infections (e.g., toxic shock syndrome and necrotizing pneumonia) [7]. This modulation of virulence expression by clindamycin occurs in clindamycin-susceptible *S.* *aureus* strains but is abolished in constitutive clindamycin-resistant strains [5]. Clindamycin resistance results from enzymatic methylation of the antibiotic binding site in the 50S ribosomal subunit (23S rRNA). The responsible methylase encoded by the *erm* gene can phenotypically result in a MLS_B_ (macrolides, lincosamides, and group B streptogramins) resistance constitutive (in vitro resistance to all MLS_B_) or inducible (a positive “D-test” in agar diffusion method: resistance to erythromycin, susceptible to lincosamides with a flattening of the inhibition zone in regard to erythromycin disc) [8]. Contrary to constitutive clindamycin-resistant strains, no data are currently available on the impact of clindamycin at sub-MICs on the virulence of inducible clindamycin-resistant *S. aureus* strains. Here, we have shown in a selection of inducible clindamycin-resistant *S. aureus* strains that clindamycin maintains its anti-toxin effect at sub-inhibitory concentrations.

## Methods

### Bacterial strain selection

One hundred eighty *S. aureus* strains from the French National Reference Centre of Staphylococci were genotyped by the *S. aureus* Genotyping Kit 2.0 (Clondiag Alere^®^ Jena, Germany), and the major macrolide resistance genes were recorded (*erm*A/B/C/T, *lin*A, *msr*A, *vat*A/B, *vga*A, *vgb,* and *mbpB*). The *mecA* gene responsible for methicillin resistance and the *lukS*-*PVL* and *tst* genes encoding for PVL and TSST-1, respectively, were also recorded.

Macrolide phenotypic resistance was determined by the agar diffusion disk assay using antibiotic discs (erythromycin, lincomycin, and quinupristin-dalfopristin) (I2A, Montpellier, France) and Mueller–Hinton E agar medium (bioMérieux, Marcy l’Etoile, France) according to the European Committee on Antimicrobial Susceptibility Testing (EUCAST) recommendations [9]. Inducible MLS_B_ resistance was detected phenotypically by an inhibition zone between the erythromycin disk and the lincomycin disks indicating a positive D-test  [8]. *S.* *aureus* strains exhibiting inducible MLS_B_ resistance were selected, and the minimal inhibitory concentrations (MIC) of clindamycin (provided by Pfizer, Amboise, France) were determined by micro-dilution method using casein hydrolysate–yeast extract medium (CCY) broth medium. The *S. aureus* strain ATCC 29213 was used for clindamycin MIC determination in both CCY (homemade) and Mueller–Hinton (bioMérieux, Marcy-l’Etoile, France) broths.

### *Staphylococcus aureus* culture conditions and toxin quantification

Since toxin production in Muller Hinton broth medium is very low, experiments were performed in CCY broth medium. Briefly, selected *S. aureus* strains were cultured overnight on Trypticase blood agar plates. Colonies were resuspended in CCY broth to a 0.5 McFarland adjusted turbidity. Cultures were performed at 37 °C with gyratory shaking (180 rpm). When the optical density reached a turbidity of a 2 McFarland (6 × 10^8^ CFU/mL), clindamycin was added to the cultures to the final concentrations of 1/2 MIC, 1/4 MIC, and 1/8 MIC. Cultures with or without clindamycin (growth control) were incubated at 37 °C with shaking for 6 h (180 rpm) [3, 10]. After the incubation time, culture supernatants were collected by centrifugation at 10,000*g* for 15 min and used for toxin quantification. The PVL, Hla and TSST-1 levels in the supernatant were quantified using a specific ELISA assay as previously described [3], using ELISA kit provided by GlaxoSmithKline, Brentford, United Kingdom, and TSST-1 antibodies provided by Toxin technology, Sarasota, FL, USA. Percentages of toxin release for each condition were calculated related to growth control without antibiotic according to formula: ${\text{\% }}\;{\text{of}}\;{\text{toxin}}\;{\text{release}} = \frac{{{\text{test}}\;{\text{with}}\;{\text{antibiotic}}}}{{{\text{growth}}\;{\text{control}}\;{\text{without}}\;{\text{antibiotic}}}} \times 100.$

## Statistical analyses

Statistical analyses were performed using RStudio, version 0.99.893 (RStudio Team (2009–2016)). RStudio: Integrated Development for R. RStudio, Inc., Boston, MA, USA). We performed t test to compare toxin expression under antibiotics conditions and growth control without antibiotics (= 100% of release).

## Results

### Inducible MLS_B_ resistance in *S. aureus* strains and the MIC of clindamycin

Among the 180 strains tested, 78 were methicillin-susceptible *S. aureus* (MSSA), and 102 were methicillin-resistant *S. aureus* (MRSA). Among these 180 strains, 112 strains harboured at least one gene encoded macrolide resistance and 68 strains harboured none. Ninety-two strains harboured only one macrolide resistance gene: 28 strains harboured *erm*A, 2 strains harboured *erm*B, 40 strains harboured *erm*C, 9 strains harboured *erm*T (all 9 belonging to the clonal complex 398), 9 strains harboured *vgaA*, 3 strains harboured *msrA* and one strain harboured *linA*. Twenty strains contained two resistance genes: 17 strains contained *msr*A and *mbp*B, one strain contained *erm*C and *lin*A, one strain contained *erm*C and *vga*A and one strain contained *msr*A and *vga*A.

Phenotypically, six strains tested positive for the D-test, indicating the presence of MLS_B_ inducible clindamycin resistance (Table 1). Three of these strains were MRSA, including a European CA-MRSA (Community-acquired MRSA) clone, ST80 PVL+ (ST2015-0934), a variant ACME negative CA-MRSA USA300 clone, ST8 PVL+ (ST2015-0940) and a Geraldine MRSA clone, ST5 TSST-1+ (ST2015-1098). The three remaining *S. aureus* strains were MSSA, including a CC-49 PVL+ strain (ST2014-0018), a CC-30 TSST-1+ strain (ST2015-0773) and a CC-121 strain (ST2014-0942). The three MRSA and the three MSSA strains, respectively, carried the *erm*C and *erm*A genes responsible for MLS_B_ inducible macrolide resistance. The MIC of clindamycin in CCY for six *S. aureus* strains was 0.25 mg/L, while the MIC of clindamycin for ATCC 29213 *S. aureus* was 0.06 mg/L in both CCY and Mueller–Hinton broth.

**Table 1:** Inducible MLS_B_ macrolide resistant strains used to explore clindamycin effect on virulence factors expression

Strain reference	Description	Clone
ST2014-0018	CC-49, *mecA*−, *ermA*+, PVL+	–
ST2015-0773	CC30, *mecA*−, *ermA*+, TSST-1 +	–
ST2014-0942	CC121, *mecA*−, *ermA*+, PVL−, TSST-1-	–
ST2015-0934	ST80, *mecA*+, *ermC*+, PVL+	European CA-MRSA
ST2015-0940	ST8, mecA+, ermC+, PVL+, ACME−	Variant USA 300 CA-MRSA
ST2015-1098	ST5, *mecA*+, *ermC*+, TSST-1+	Geraldine

*CA-MRSA* community-acquired methicillin-resistant *Staphylococcus aureus*

### Effect of clindamycin on Hla expression and release in MLS_B_ inducible *S. aureus* strains

According to culture condition, means of Hla measurements (two determinations) in growth control supernatant were equal to 60.96, 52.00, 39.84, 19.79, 3.23 and < 0.75 ng/mL for ST2014-0018, ST2015-0940, ST2015-0934, ST2014-0942, ST2015-1098 and ST2015-0773 respectively.

**Fig. 1:**
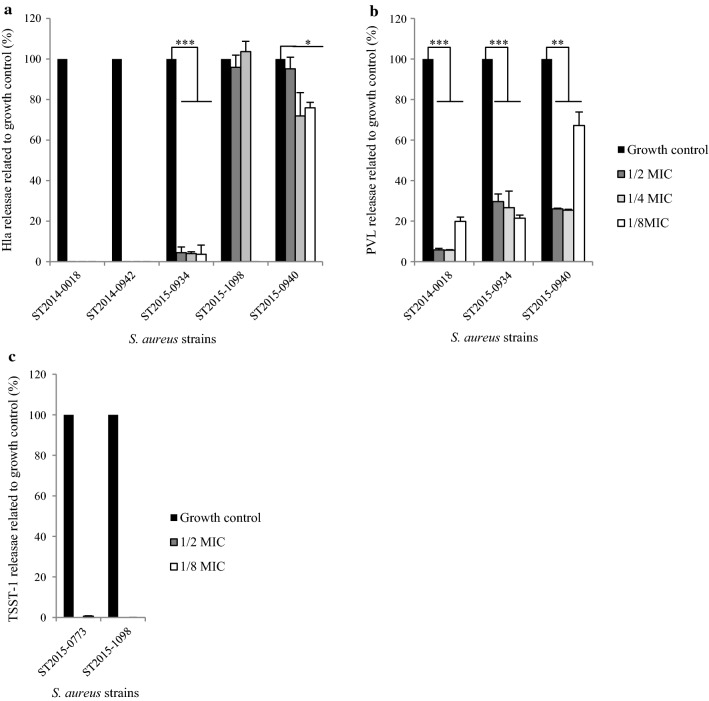
Virulence factors release by inducible MLS_B_-resistant *S. aureus* strains after sub-MIC of clindamycin treatment. Hla (**a**), PVL (**b**) and TSST-1 (**c**) release were measured by ELISA detection after clindamycin treatment (1/2 MIC, 1/4 MIC and 1/8 MIC) in comparison to the growth control without clindamycin. The averages represent two determinations for each condition. For each strain, virulence factor release at 1/2 MIC, 1/4 MIC and 1/8 MIC of clindamycin were pooled (n = 6) to perform the t test to compare with growth control without antibiotic (= 100%) and calculate the 95% confidence interval of the average. *p < 0.05; **p < 0.01; ***p < 0.001. Lack of a chart bar means that the factor virulence release measure was below the detection limit (i.e., 0.75 ng/mL for Hla and 1.56 ng/mL for TSST-1)

Sub-MICs of clindamycin (from 1/2 MIC to 1/8 MIC) induced a dramatic decrease of supernatant Hla for three strains (ST2014-0018, ST2014-0942 and ST2015-0934) ranging from undetectable Hla levels for ST2014-0018 and ST2014-0942 to low Hla levels (4.07%, CI_95%_ 1.53, 6.62, p < 0.001) for ST2015-0934 at each clindamycin concentration (Fig. 1a). For ST2015-1098, only 1/8 MIC of clindamycin suppressed Hla production (undetectable level), and for ST2015-0940, we observed a slight significant decrease in Hla production (80.99%, CI_95%_, 67.81, 94.17, p < 0.05) at 1/4 MIC and 1/8 MIC of clindamycin (Fig. 1a). For the ST2015-0773 strain, Hla was undetectable by the ELISA assay regardless of the experimental conditions (control or with antibiotic) most likely due to a truncated Hla variant present in CC-30 strains, which the ELISA assay was unable to detect [11].

### Effect of clindamycin on PVL expression and release in MLS_B_ inducible *S. aureus* strains

According to culture condition, means of PVL measurements (two determinations) in growth control supernatant were equal to 682.43, 327.30 and 161.89 ng/mL for ST2015-0940, ST2015-0934 and ST2017-0018 respectively.

Of the six *S. aureus* strains tested, three stains carried the *lukS*-*PVL* gene. For these three strains, the sub-MICs of clindamycin significantly decreased PVL release compared to that of the growth control (without antibiotics) (Fig. 1b). PVL concentrations, expressed as percentages of the growth control levels, in the supernatants treated with clindamycin at sub-MICs ranged from 10.46% (CI_95%_, 2.74, 18.18, p < 0.01) for ST2014-0018, 25.93% (CI_95%_, 20.15, 31.71, p < 0.001) for ST2015-0934, and 39.55% (CI_95%_, 16.87, 62.23, p < 0.01) for ST2015-0940.

### Effect of clindamycin on TSST-1 expression and release in MLS_B_ inducible *S. aureus* strains

Of the six *S. aureus* strains tested, two carried the *tst* gene and exhibited variable TSST-1 expression in comparison to that of the growth control: 251.04 ng/mL for ST2015-0773 and 2.49 ng/mL for ST2015-1098. The sub-MICs of clindamycin induced a dramatic decrease of TSST-1 release for ST2015-0773 with an undetectable TSST-1 expression at 1/2 MIC of clindamycin and an approximate 99% decrease at 1/8 MIC. For ST2015-1098, 1/2 MIC and 1/8 MIC of clindamycin resulted in an undetectable TSST-1 expression level (Fig. 1c).

## Discussion and conclusions

*Staphylococcus aureus* produces many virulence factors that play an important role in the pathogenesis of infection, such as Hla [12], PVL [13, 14] and TSST-1 [15]. Several studies have shown that clindamycin displays an anti-toxin in vitro effect at sub-inhibitory concentrations, inducing a decrease in toxin release [1–6]. Moreover, in a rabbit model of PVL+ CA-MRSA-induced necrotizing pneumonia, clindamycin was superior to vancomycin in reducing PVL induced tissue damage and the overall mortality rate, consistent with decreased PVL pulmonary concentrations [16]. Several case reports of necrotizing pneumonia and staphylococcal toxic shock highlight clindamycin’s efficacy when used as an adjunctive antibiotic for the treatment of *S. aureus* related toxin infections [17]. Notably, a retrospective analysis of 92 cases of CA-MRSA necrotizing pneumonia, mainly due to PVL-producing strains, showed improved clinical outcomes in patients treated with antimicrobials inhibiting toxin production (linezolid or clindamycin) [18]. The underlying mechanism of clindamycin’s anti-toxin effect is linked to the ribosome-blocking action in which the transcription of exoprotein and SaeRS global regulator system, are reduced [5, 19]. This anti-toxin effect is abolished by the constitutive MLS_B_ resistance mechanism [5] in macrolide and lincosamide-resistant strains.

Clindamycin resistance can be constitutive or inducible. *S. aureus* strains belonging to the latter category display, when tested, in vitro susceptibility to clindamycin. Nevertheless, in the first decade of the 21st century, treatment failure was reported in adult and paediatric populations when clindamycin was used for inducible MLS_B_ *S. aureus* infections [8, 20]. Consequently, the Clinical and Laboratory Standards Institute and EUCAST recommend searching and reporting for the inducible MLS_B_ phenotype, but also stating that clindamycin may still be used for short-term therapy [9, 20].

Nevertheless, no data are currently available on the anti-toxin effect of clindamycin in inducible MLS_B_-resistant *S. aureus* isolates. Here, we showed that sub-MICs of clindamycin efficiently resulted in a decrease in PVL, Hla and TSST-1 production for inducible MLS_B_ *S. aureus* strains in vitro, regardless of the methicillin susceptibility. For one strain, ST2015-1098, displaying a very weak level of Hla expression in control condition (3.23 ng/mL), we detected suppressed Hla production only 1/8 MIC of clindamycin. We suspected a lack of sensitivity of the measure method which could have failed to detect Hla decreased expression at 1/2 and 1/4 MIC of clindamycin, explaining the discrepancies between antibiotics concentrations. Interestingly, we observed that the decrease of virulence factor release (Hla and PVL) in the presence of clindamycin at sub-MICs was less altered for the variant USA300 clone (ST2015-0940). We hypothesize that this effect is likely dependent on the genetic background. This observation is in agreement with the study of Martin-Cardot et al., which had shown that the USA300 clone was resistant to staphylococcal protein A expression modulation by antibiotics and antimicrobial peptides [10].

In this short report, we chose to focus on the phenotypic expression of toxins, with possible direct impact on the clinical management of infections related to staphylococcal toxins, and without transcriptomic data because we and other authors, previously observed that modification at transcriptomic level did not always reflect modulation of toxins production [17].

In conclusion, our results indicate that the anti-toxin effect of clindamycin should therefore be considered when treating toxin related infections for inducible clindamycin-resistant *S. aureus*. However, given the risk of constitutive MLS_B_ resistance acquisition upon clindamycin treatment, clindamycin should be coupled with a bactericidal effective antibiotic for the treatment of toxin-related *S. aureus* severe infections.

## Abbreviations

CA-MRSA: community-acquired methicillin-resistant *Staphylococcus aureus*
EUCAST: European Committee on Antimicrobial Susceptibility Testing
Hla: alpha-haemolysin
MIC: minimal inhibitory concentration
MLS_B_: macrolides, lincosamides, and group B streptogramins
MRSA: methicillin-resistant *Staphylococcus aureus*
MSSA: methicillin-susceptible *Staphylococcus aureus*
PVL: Panton–Valentine leucocidin
Sub-MICs: sub-inhibitory concentrations
TSST-1: toxic-shock-staphylococcal toxin
